# Periodic sharp wave triplets and quadruplets

**DOI:** 10.4103/0972-2327.40231

**Published:** 2008

**Authors:** C. J. Suresh Chandran

**Affiliations:** Department of Neurology, Kerala Institute of Medical Sciences, Trivandrum, Kerala, India

A 65-year-old male presented with acute inferior wall myocardial infarction and cardiogenic shock. In the intensive coronary care unit, he developed cardiac arrest. He was resuscitated and was put on ventilatory and inotropic support. On examination, he was deeply comatos (Glasgow Coma Scale E_1_M_2_V_ET_), pupils were mid-dilated and sluggishly reaction and oculocephalic reflex was absent. Occasional spontaneous respiratory efforts were noted. No myoclonic jerks were observed. A diagnosis of hypoxic ischemic encephalopathy (HIE) was made.

**Figure 1:**
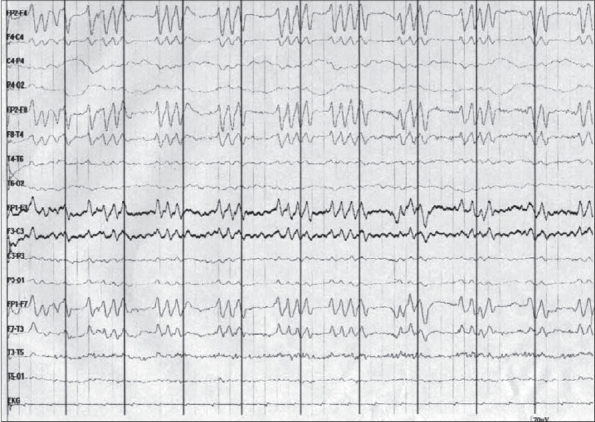
EEG showing periodic paroxysms of sharp wave triplets and quadruplets with attenuation of activity between the complexes

**Figure 2:**
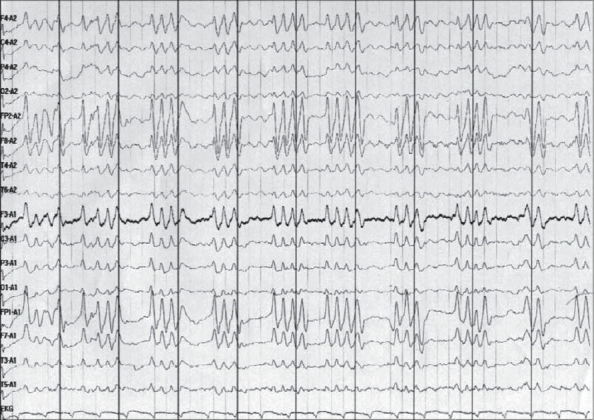
Generalized periodic polysharp wave paroxysms with a duration of 0.5 to 0.8 s, occurring at a periodicity of 0.3-0.5 s. The complexes show bifrontal predominance

A bedside electroencephalogram was performed one hour after the cardiopulmonary arrest and showed generalized periodic sharp waves in clusters of triplets and quadruplets with attenuation of background activity between the complexes [Figures 1 and 2]. The duration of individual polysharpwave paroxysms varied from 0.5 to 0.8 s, and they occurred at a periodicity of 0.3 to 0.5 s. The sharp wave triplets and quadruplets showed an anteroposterior gradient. No change was noted in the EEG after the administration of lorazepam (4 mg) intravenously. The patient developed refractory shock and expired 3 h after the cardiopulmonary arrest.

Electroencephalogram (EEG) in post anoxic coma shows several patterns, the most severe being complete generalized suppression.[1,2] Several other patterns such as less marked suppression, burst-suppression, dominant theta-delta activity, epileptiform activity, periodic complexes and alpha coma patterns are also observed.[1–3] Periodic complexes on EEG may have various morphologies that recur at regular intervals in routine EEG, and generalized periodic bisynchronous sharp waves have previously been described in a variety of toxic, metabolic or degenerative (i.e., CJD) encephalopathies in addition to hypoxia and status epilepticus.[3] The EEG monitoring in an intensive care unit is important as particular patterns have diagnostic and prognostic significance (e.g., spindle coma, alpha coma, burst-suppression activity and triphasic waves).[4] Periodic generalized phenomena in postanoxic coma carry a poor prognosis.[1] The periodic sharp wave triplets and quadruplets in this case appear to be due to severe bilateral hemispheric injury. Nonconvulsive status epilepticus also merits consideration as the periodic polysharp wave frequency is more than 3/s, and the duration of the individual paroxysms is greater than 0.5s.[5] However, there was no change in these complexes after administering lorazepam. Unfortunately, we could not obtain prolonged recording or repeat EEGs. Further definition and the significance of this reported feature must be elucidated more completely with prolonged EEG monitoring or serial tracings.
